# Comparison between single and dual antiplatelet therapy in patients on oral anticoagulants undergoing coil embolization for unruptured intracranial aneurysms: a retrospective multicenter cohort study

**DOI:** 10.1007/s00234-025-03844-2

**Published:** 2025-11-18

**Authors:** Seung Pil Ban, O-Ki Kwon, Young Deok Kim, Hwan Seok Shim, Seung Bin Sung, Jung Cheol Park, Hyoung Soo Byoun, Min Jai Cho, Hyunjun Jo, Hyun Park, Sukh Que Park, Dongwook Seo, Jang Hun Kim, Yu Deok Won, Seunghyun Won

**Affiliations:** 1 Department of Neurosurgery, Seoul National University Bundang Hospital, Seongnam-si, Republic of Korea; 2 Department of Neurosurgery, Seoul National University College of Medicine, Seoul, Republic of Korea; 3 Department of Neurosurgery, Asan Medical Center, Seoul, Republic of Korea; 4 Department of Neurosurgery, Chungnam National University Sejong Hospital, Sejong, Republic of Korea; 5 Department of Neurosurgery, Chungbuk National University Hospital, Cheongju-si, Republic of Korea; 6 Department of Neurosurgery, Korea University Guro Hospital, Seoul, Republic of Korea; 7 Department of Neurosurgery, Gyeongsang National University Hospital, Jinju, Republic of Korea; 8 Department of Neurosurgery, Soonchunhyang University Seoul Hospital, Seoul, Republic of Korea; 9 Department of Neurosurgery, Korea University Anam Hospital, Seoul, Republic of Korea; 10 Department of Neurosurgery, Hanyang University Guri Hospital, Guri, Republic of Korea; 11 Division of Statistics, Medical Collaborating Research Center, Seoul National University Bundang Hospital, Seongnam-si, Republic of Korea

**Keywords:** Cerebral aneurysm, Anticoagulant, Antiplatelet, Hemorrhage, Thrombosis

## Abstract

**Purpose:**

The optimal antiplatelet therapy for patients on continuous oral anticoagulants (OACs) who are undergoing coil embolization for unruptured intracranial aneurysms (UIAs) is remains unknown. This study evaluated the efficacy and safety of single- (SAPT) and dual antiplatelet therapy (DAPT) in patients taking OACs who underwent coil embolization for UIAs.

**Methods:**

This retrospective multicenter study included patients taking OACs who underwent coil embolization for UIAs at 9 hospitals between January 2016 and August 2023. The primary outcome was a thromboembolic complication within 30 days post-procedure. The secondary outcome was a composite all bleeding events according to the Thrombolysis in Myocardial Infarction bleeding criteria.

**Results:**

A total of 112 patients (mean [standard deviation] age, 67.3 [9.7]; 67 females [59.8%]) were included. Among them, 31 patients (27.7%) received SAPT, and 81 patients (72.3%) received DAPT. There was no significant difference in the thromboembolic event rate between the 2 groups (SAPT group: 2 of 31 [6.5%]; DAPT group: 3 of 81 [3.7%]; unadjusted hazard ratio [HR], 0.55 [95% CI, 0.09–3.30]; *P* = .52). However, the rate of all bleeding events after coil embolization in the DAPT group was significantly higher than that in the SAPT group (SAPT group: 2 of 31 [6.5%]; DAPT group: 22 of 81 [27.2%]; adjusted HR, 5.57 [95% CI, 1.30-23.83]; *P* = .02).

**Conclusions:**

With respect to SAPT, DAPT was not associated with a reduction in thromboembolic complications in patients taking OACs who underwent coil embolization, but it was associated with an increase in all bleeding events.

**Supplementary Information:**

The online version contains supplementary material available at 10.1007/s00234-025-03844-2.

## Introduction

Dual antiplatelet therapy (DAPT), typically consisting of aspirin and clopidogrel, is commonly administered to prevent thromboembolic complications in patients undergoing coil embolization for unruptured intracranial aneurysms (UIAs) [1]. Recently, the use of oral anticoagulants (OACs) has increased, given their efficacy in the management of conditions such as atrial fibrillation, valvular heart disease, and other thromboembolic diseases [2]. Therefore, there is a high likelihood that coil embolization procedures are being performed on patients taking OACs.

In this context, physicians face substantial difficulties in determining the optimal antithrombotic regimen that best balances safety and efficacy for patients undergoing coil embolization. Warfarin or direct oral anticoagulants (DOACs) are necessary to prevent stroke in patients with underlying conditions that pose a high risk of embolic stroke. Although switching to DAPT may be a more effective option for reducing stroke related to coil embolization, discontinuing OACs for patients undergoing coil embolization increases the risk of thromboembolic events associated with the underlying conditions [3]. If the OAC and DAPT are administered concurrently, the increased antithrombotic effect may reduce thromboembolic complications; however, it may also increase the risk of hemorrhagic complications [4]. Previous studies have reported an increased risk of hemorrhage during coil embolization or flow diverter treatment of UIAs in patients taking OACs [5–7]. However, these studies included only on the order of ten patients, and there are no established guidelines for the appropriate combination of OAC and antiplatelet therapy for patients on continuous OACs who are undergoing coil embolization for UIAs. Therefore, identifying an optimal antiplatelet therapy regimen that can reduce both thromboembolic and hemorrhagic complications during coil embolization in patients maintaining OACs is essential.

We conducted a retrospective multicenter cohort study to evaluate the efficacy and safety of two antiplatelet therapy regimens, single antiplatelet therapy (SAPT) and DAPT, in patients taking OACs who underwent coil embolization for UIAs.

## Materials and methods

### Study design and participants

This retrospective, multicenter, comparative cohort study was conducted according to the recommendations of the Strengthening the Reporting of Observational Studies in Epidemiology reporting guidelines. This study was approved by the institutional review board (IRB) of Seoul National University Bundang Hospital, and each participating institution obtained approval from their respective local IRBs. Given the retrospective and blinded nature of the study, informed consent of patients was waived.

The study cohort included individuals aged 20 years or older who underwent coil embolization for UIAs at 9 hospitals between January 1, 2016, and August 31, 2023 (Table 1).

**Table 1:** Participating institutions and number of enrolled patients

Participating institution (Total, 9 institutions)	Number of patients who underwent coil embolization for UIAs (Total, *n* = 11308)	Number of included patients (Total, *n* = 112)	Procedural period
1	5084	53	January/2016 – August/2023
2	3409	29	January/2016 – August/2023
3	72	3	July/2020 – August/2023
4	134	2	January/2018 – August/2023
5	429	7	January/2016 – August/2023
6	210	1	January/2016-August/2023
7	1278	7	January/2016 – August/2023
8	159	7	June/2020 – August/2023
9	533	3	January/2016-August/2023

*UIA* unruptured intracranial aneurysm

The inclusion criteria were as follows: (1) coil embolization with or without a stent for UIAs, (2) continuous OAC due to underlying diseases such as atrial fibrillation, valvular heart disease, deep vein thrombosis, pulmonary embolism, and cerebrovascular disease, and (3) SAPT or DAPT administration for at least 5 days before the procedure without discontinuing OAC use. Patients who were treated with flow diverters or intrasaccular devices and those who received triple antiplatelet therapy were excluded.

Age, sex, past history, medical history, medication for underlying diseases, laboratory data, aneurysmal data, OAC data, procedural data, and data on antiplatelet regimens were obtained by reviewing the electronic medical records (EMRs) and radiological images of the patients. Patients were assigned to either the SAPT group or the DAPT group on the basis of the data obtained from their EMRs. The SAPT group was defined as those patients on OACs and who received either aspirin or clopidogrel as an antiplatelet single therapy, whereas the DAPT group was defined as patients on OACs and who received both aspirin and clopidogrel. All coil embolization procedures were performed under general anesthesia. Detailed on the coil embolization procedures are presented in Supplemental Method 1.

### Outcomes

The primary outcome was a thromboembolic complication during the periprocedural period (within 30 days after coil embolization), defined as the occurrence of thromboembolism during coil embolization or a transient ischemic attack (TIA) or ischemic stroke with evidence of infarction on diffusion-weighted imaging that was localized to a vascular territory associated with the site of the procedure [8, 9]. We differentiated TIA from ischemic stroke according to symptom duration [10].

The secondary outcome was a composite outcome of all bleeding events (major, minor or minimal bleeding), defined according to the Thrombolysis in Myocardial Infarction bleeding criteria, within 30 days after coil embolization [11].

### Statistical analysis

Baseline characteristics were compared using the Wilcoxon rank-sum test for continuous variables and the χ^2^ test or Fisher exact test for categorical variables, as appropriate. The cumulative incidences of thromboembolic and bleeding events in the SAPT and DAPT groups were estimated with the Kaplan-Meier method, and differences between groups were evaluated with the log-rank test. Univariable Cox proportional hazards regression was performed to identify potential predictors of each outcome; variables with *P* <.20 in the univariable analyses were entered into a multivariable Cox model. Firth’s penalized likelihood estimation was applied in the Cox regression to mitigate convergence issues arising from sparse event data and separation. All the statistical analyses were performed in R version 4.2.2 (R foundation for Statistical Computing). All tests were two-sided, and *P* <.05 was considered to indicate statistical significance.

## Results

### Study participants and characteristics

**Fig. 1:**
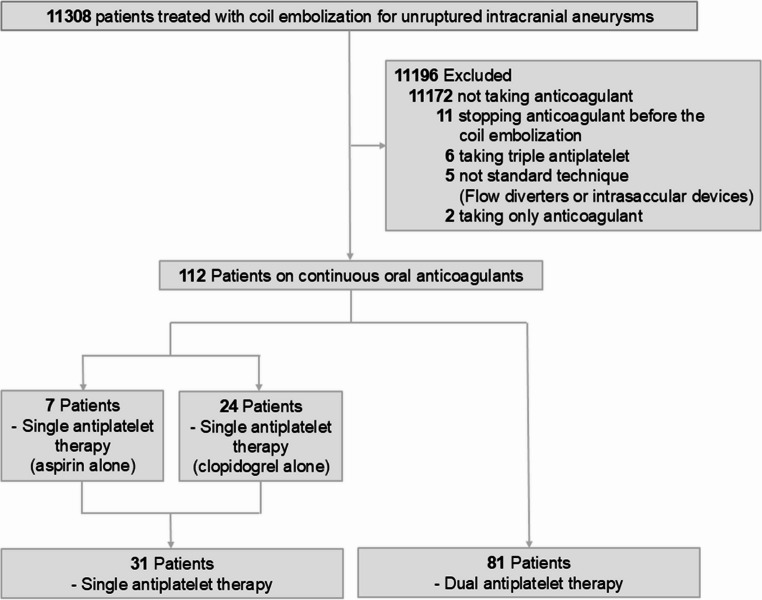
Flow chart of the study participants and their antithrombotic medications

Between January 1, 2016, and August 31, 2023, 11,308 patients across 9 institutions were treated with coil embolization for UIAs. Among these patients, 11,172 were not taking OACs, 11 discontinued OAC use prior to the procedure, 6 patients received triple antiplatelet therapy, 5 patients were treated with flow diverters or intrasaccular devices instead of standard coil embolization, and 2 patients were only taking OACs. Therefore, a total of 11,196 patients were excluded, and the final study population included 112 patients (mean [standard deviation] age, 67.3 [9.7]; 45 males [40.2%] and 67 females [59.8%]). Among 112 patients, 31 patients (27.7%) received SAPT (aspirin alone, 7 patients [20.6%]; clopidogrel alone, 24 patients [77.4%]), whereas 81 patients (72.3%) received DAPT (Fig. 1). There was no loss to follow-up during the 1-month follow-up after coil embolization.

A comparison of the baseline characteristics of patients in both antiplatelet therapy groups is shown in Table 2. Except for a history of alcohol consumption (SAPT group, 2 of 31 [6.5%]; DAPT group, 20 of 81 [24.7%]; *P* =.03), the baseline characteristics of the SAPT and DAPT groups were well balanced. Coil embolization was successfully performed for all 112 patients, and among them, 59 (52.7%) underwent stent-assisted coil embolization. Among the study patients, 22 took warfarin, and 90 took DOACs (edoxaban, 30 of 90 [33.3%]; apixaban, 28 of 90 [31.1%]; rivaroxaban, 26 of 90 [28.9%]; dabigatran, 6 of 90 [6.7%]). The most common indication for OAC use was atrial fibrillation (*n* = 74, 66.1%), followed by valvular heart disease (*n* = 20, 17.9%), deep vein thrombosis (*n* = 8, 7.1%), pulmonary embolism (*n* = 6, 5.3%), and cerebrovascular disease (*n* = 4, 3.6%).

**Table 2:** Baseline characteristics

Characteristic	Patients, No. (%)				
	Single antiplatelet therapy group, aspirin alone (*n* = 7)	Single antiplatelet therapy group, clopidogrel alone (*n* = 24)	Single antiplatelet therapy group, overall (*n* = 31)	Dual antiplatelet therapy group (*n* = 81)	*p*-value^*^
Age, mean (SD) y	70.4 (9.1)	66.2 (9.2)	67.2 (9.2)	67.4 (10.0)	0.91
Female sex	6 (85.7)	16 (66.7)	22 (71.0)	45 (55.6)	0.20
Smoking	0 (0.0)	5 (20.8)	5 (16.1)	21 (25.9)	0.33
Drinking	0 (0.0)	2 (8.3)	2 (6.5)	20 (24.7)	0.03
Medical history					
Hypertension	5 (71.4)	20 (83.3)	25 (80.6)	70 (86.4)	0.56
Dyslipidemia	3 (42.9)	15 (62.5)	18 (58.1)	45 (55.6)	0.84
Diabetes	2 (28.6)	1 (4.2)	3 (9.7)	22 (27.2)	0.07
Stroke	0 (0.0)	6 (25.0)	6 (19.4)	20 (24.7)	0.63
Laboratory data, mean (SD)					
Platelet count, ×10^3^/㎕	255.5 (48.4)	198.9 (48.4)	207.5 (68.8)	232.1 (87.5)	0.10
aPTT, s	56.7 (25.8)	40.2 (7.8)	43.7 (24.0)	39.7 (22.7)	0.39
PT, INR	1.6 (0.4)	1.5 (0.4)	1.5 (0.4)	1.3 (0.5)	0.07
Total cholesterol level, mg/dL	157.5 (29.0)	157.7 (31.7)	157.7 (30.8)	154.4 (41.8)	0.63
Aneurysm data					
Maximum size, mean (SD), mm	5.9 (4.0)	5.3 (2.7)	5.4 (3.0)	6.0 (2.4)	0.29
Neck size, mean (SD), mm	4.3 (2.4)	3.3 (1.4)	3.5 (1.7)	4.1 (1.6)	0.06
Dome-to-neck ratio, mean (SD)	1.3 (0.3)	0.8 (0.5)	0.9 (0.5)	1.1 (0.5)	0.05
Location					0.08
ACA	2 (28.6)	4 (16.7)	6 (19.4)	34 (42.0)	
ICA	3 (42.9)	8 (33.3)	11 (35.5)	26 (32.1)	
MCA	1 (14.3)	8 (33.3)	9 (29.0)	11 (13.6)	
Posterior circulation	1 (14.3)	4 (16.7)	5 (16.1)	10 (12.3)	
Oral anticoagulant data					
Oral anticoagulant					0.43
Warfarin	3 (42.9)	5 (20.8)	8 (25.8)	14 (17.3)	
DOAC	4 (57.1)	19 (79.2)	23 (74.2)	67 (82.7)	
Indication of oral anticoagulant					0.49
Atrial fibrillation	2 (28.6)	17 (70.8)	19 (61.3)	55 (67.9)	
Valvular heart disease	2 (28.6)	5 (20.8)	7 (22.6)	13 (16.1)	
Deep vein thrombosis	0 (0.0)	1 (4.2)	1 (3.2)	7 (8.6)	
Pulmonary embolism	3 (42.8)	0 (0.0)	3 (9.7)	3 (3.7)	
Cerebrovascular disease	0 (0.0)	1 (4.2)	1 (3.2)	3 (3.7)	
Procedural data					
Coiling					0.83
Non-stent-assisted	3 (42.9)	11 (45.8)	14 (45.2)	39 (48.1)	
Stent-assisted	4 (57.1)	13 (54.2)	17 (54.8)	42 (51.9)	
Systematic heparinization					0.62
No	2 (28.6)	6 (25.0)	8 (25.8)	17 (21.0)	
Yes	5 (71.4)	18 (75.0)	23 (74.2)	64 (79.0)	
Packing density, %^†^	40.3 (12.4)	42.3 (11.5)	41.9 (12.5)	38.2 (12.6)	0.26
Occlusion grade^‡^					0.70
Complete	5 (71.4)	19 (79.1)	24 (77.5)	63 (77.8)	
Residual neck	2 (28.6)	4 (16.7)	6 (19.3)	16 (19.7)	
Residual sac	0 (0.0)	1 (4.2)	1 (3.2)	2 (2.5)	

*ACA* anterior cerebral artery, *aPTT* activated partial thromboplastin time, *DOAC* direct oral anticoagulant, *ICA* internal carotid artery, *INR* international normalized ratio, *MCA* middle cerebral artery, *PT* prothrombin time, *SD* standard deviation

^*^The P value measured the comparison between the overall single antiplatelet therapy group and the dual antiplatelet therapy group.

^*†*^Aneurysm volume was measured with the Vitrea Advanced Visualization software (version 6.9.68.1, Vital Images) and coil volume was calculated online (http://www.angiocalc.com). Using these 2 volumes, packing density (%, coil/aneurysm volume × 100) was obtained.

^*‡*^Evaluated according to the Raymond grade.

### Primary and secondary outcome

**Fig. 2:**
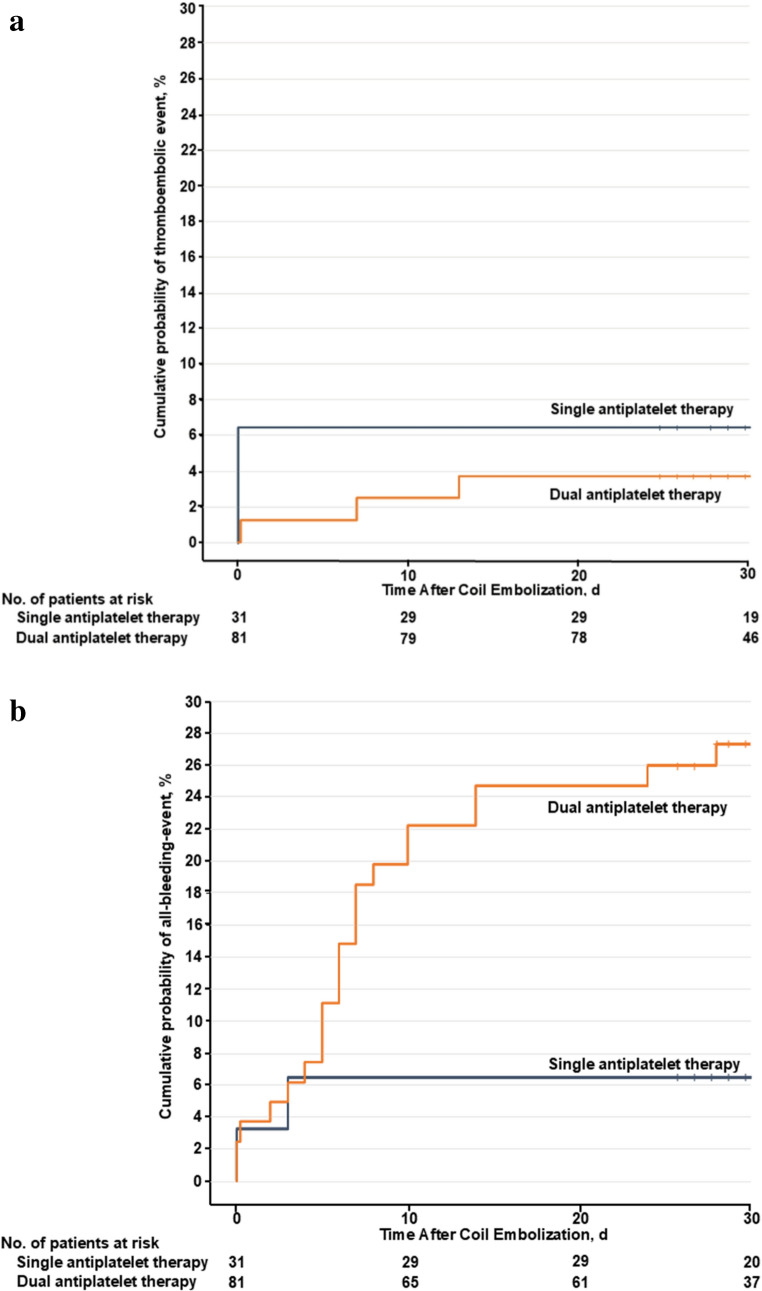
Kaplan-Meier Curves for the Single and Dual Antiplatelet Therapy Groups. (**A**) Thromboembolic events and (**B**) All bleeding events

Thromboembolic complications developed in 5 patients (4.5%) and were detected during the procedure or within 30 days after coil embolization (Supplemental Table 1). The thromboembolic event rate during the procedure or within the 30-day follow-up period after procedure did not differ between the 2 groups (SAPT group: 2 of 31 [6.5%]; DAPT group: 3 of 81 [3.7%]; unadjusted hazard ratio [HR], 0.55 [95% CI, 0.09–3.30]; *P* =.52; Table 3; Fig. 2A and Supplemental Fig. 1 A). There was no significant difference in the thromboembolic complication rate among the 3 groups: aspirin alone (1 of 7 [14.3%]), clopidogrel alone (1 of 23 [4.2%]; unadjusted HR, 0.27 [95% CI, 0.02–4.32]; *P* =.36), and aspirin with clopidogrel (3 of 81 [3.7%]; unadjusted HR, 0.24 [95% CI, 0.02–2.26]; *P* =.21) (Supplemental Fig. 2). The thromboembolic event rates by OAC type were 0% (0 of 22) in the warfarin group and 5.6% (5 of 90) in the DOAC group.

**Table 3:** Primary and secondary outcomes

Event	Events, No. (%)						
	Single antiplatelet therapy group, aspirin alone (*n* = 7)	Single antiplatelet therapy group, clopidogrel alone (*n* = 24)	Single antiplatelet therapy group, overall (*n* = 31)	Dual antiplatelet therapy group, aspirin with clopidogrel (*n* = 81)	Unadjusted Hazard Ratio (95% CI)	Adjusted^*^ Hazard Ratio (95% CI)	*P* Value^†^
Thromboembolic events	1 (14.3)	1 (4.2)	2 (6.5)	3 (3.7)	0.55 (0.09–3.30)	Not available	0.52
Bleeding events, all^‡^	0 (0.0)	2 (8.3)	2 (6.5)	22 (27.2)	4.57 (1.07–19.4)	5.57 (1.30–23.83.30.83)	0.02
Bleeding events, major^‡^	0 (0.0)	1 (4.2)	1 (3.2)	2 (2.5)	0.76 (0.07–8.39)	Not available	0.65
Bleeding events, minor/minimal^‡^	0 (0.0)	1 (4.2) (	1 (3.2)	20 (24.7)	8.41 (1.13–62.65)	10.44 (1.40–78.00)	0.02

^*^Secondary outcome comparison was adjusted for smoking status, alcohol consumption, heparin administration, the presence of hypertension, maximum aneurysm diameter, and the use of a stent-assisted technique. Multivariable analysis for thromboembolic events was not feasible because of the low number of events.

^†^The *P* value measured the comparison between the overall single antiplatelet group and the dual antiplatelet group.

^‡^Evaluated according to the Thrombolysis in Myocardial Infarction bleeding criteria.

Bleeding events occurred in 24 patients (21.4%) within the 30-day follow-up period (Supplemental Table 2). The rate of all bleeding events after coil embolization in the DAPT group was significantly higher than that in the SAPT group (SAPT group: 2 of 31 [6.5%]; DAPT group: 22 of 81 [27.2%]; unadjusted HR, 4.57 [95% CI, 1.07–19.4]; adjusted HR, 5.57 [95% CI, 1.30–23.83.30.83]; *P* =.02; Table 3; Fig. 2B and Supplemental Fig. 1B). The major bleeding event rate during the procedure or within the 30-day follow-up period after procedure did not differ between the 2 groups (SAPT group: 1 of 31 [3.2%]; DAPT group: 2 of 81 [2.5%]; unadjusted HR, 0.76 [95% CI, 0.07–8.39]; *P* =.65; Table 3). There was no significant difference in the all-bleeding event rate between the groups based on the type of OAC used (warfarin group: 1 of 22 [4.5%]; DOAC group: 23 of 90 [25.6%]; unadjusted HR, 6.26 [95% CI, 0.85–46.4]; adjusted HR, 5.55 [95% CI, 0.71–41.31]; *P* =.09).

## Discussion

Both ischemic and hemorrhagic complications are major concerns for patients undergoing coil embolization. This is of particularly concern for patients who are already taking medications that impose high bleeding risks, such as OACs. In this study, we found no significant difference in the thromboembolic event rate between the SAPT group and the DAPT group within the 30-day follow-up period after coil embolization for UIAs. However, the risk of all bleeding events was greater in the DAPT group than in the SAPT group.

According to American Heart Association guidelines, patients with chronic coronary disease (CCD) who have undergone elective percutaneous coronary intervention (PCI) and who require OAC therapy should be administered DAPT for 1 to 4 weeks followed by clopidogrel alone for 6 months in addition to DOAC [12]. However, there are limitations in applying the same antiplatelet therapy used for coronary disease, which has a strong ischemic nature due to the associated atherosclerotic burden, to coil embolization for UIAs. Although there are no clear antiplatelet therapy guidelines for neurointerventional procedures for these patients, the 2023 Society of NeuroInterventional Surgery (SNIS) guideline update provides a generally similar approach [13]. According to the SNIS guidelines, in patients with UIAs who are receiving OAC for atrial fibrillation or acute systemic venous thrombosis, it is recommended to administer OAC plus SAPT or OAC plus DAPT. The management of these patients should consider both thrombotic and hemorrhagic risks, and these risks should be reassessed after 3 months to inform modification of the regimen. However, these guidelines are not based on neurointerventional data, and antithrombotic treatment for patients undergoing coil embolization for UIAs is recommended not as an exhaustive approach but rather to be individualized to each patient through multidisciplinary management. Furthermore, the few studies related to OAC therapy in the context of aneurysms have been limited to comparisons between antiplatelet therapy alone and OAC plus antiplatelet therapy [5–7]. Therefore, the optimal antiplatelet therapy for patients receiving OAC in neurointerventional treatment remains unknown. Similar to previous CCD studies [14–16], this study revealed no difference in the risk of thromboembolic complications between the SAPT and DAPT groups. Even when SAPT was further classified into aspirin alone and clopidogrel alone therapies, there was no difference in the thromboembolic complication rate between those groups. This contrasts with the findings of studies on coronary disease patients, which have suggested that clopidogrel alone is associated with a lower thromboembolic risk than aspirin alone [17]. This discrepancy is likely due to the small number of aspirin alone therapy patients and the short follow-up period in this study. Although statistically insignificant, the aspirin alone therapy group demonstrated a greater occurrence (14.3%) of thromboembolic events than the other groups in this study. In a study involving antithrombotic treatment for carotid stenting in patients with concomitant atrial fibrillation, similar to our findings, no differences in thromboembolic events were observed in the groups receiving OAC plus clopidogrel, OAC plus DAPT, or DAPT alone [18]. Although limited by a sample size of only 91 patients, the study demonstrated similar results in the context of neurointerventional treatment, thereby providing evidence for the effectiveness of OAC plus SAPT.

Hemorrhagic complications are also a major concern when antithrombotic therapy is used. In this study, similar to the findings of previous coronary disease studies, the rate of all bleeding events was higher in the DAPT groups. However, the bleeding rate in this study could be seen as very low. For example, the bleeding rate in the What is the Optimal antiplatElet and anticoagulant therapy in patients with oral anticoagulant and coronary StenTing (WOEST) trial among 573 patients treated with PCI was notably higher at 1 year in the SAPT (19.4%) and DAPT groups (44.4%) [14]. This difference may be attributed to the shorter follow-up period of this study (1 month) than that of the WOEST trial (1 year), despite our study including all bleeding events. Consistent with this study, in a study with a one-month follow-up after carotid stenting [18], similar to our findings, the OAC plus DAPT group had a significantly higher incidence of all bleeding events (23.8% [9.5% major bleeding]), whereas the incidence was 4% in the DAPT group and absent in the OAC plus SAPT group. Since the risk of bleeding complications increases with longer use of multiple antithrombotic therapy [15, 16], the shorter follow-up period in this study may have contributed to the lower observed bleeding rate. it appears well established that combining DAPT with OACs increases the risk of bleeding; therefore, when DAPT is necessary, considerable caution is needed, and the duration of DAPT should be kept as short as possible.

In patients on OACs undergoing coil embolization for UIAs, one might consider discontinuing OAC use and implementing the standard approach of DAPT for coil embolization treatment. Among the 11,308 patients initially recruited for this study, only 11 were in this situation, but the impact of this strategy was not analyzed. According to a coronary study [19], in patients with atrial fibrillation, full-intensity OACs provided better protection against ischemic complications than did DAPT with aspirin and clopidogrel. In addition, withholding OACs in atrial fibrillation patients undergoing PCI has been associated with an increased risk of major cardiovascular events and mortality while having no effects on reducing the risk of bleeding [20]. Additionally, omitting clopidogrel in PCI patients has been linked to a higher risk of thromboembolic events [21]. Therefore, the most appropriate option for these patients is the combination of OACs with clopidogrel. According to the findings of this study, and similar to the conclusions of coronary studies [12, 16], in patients on continuous OACs undergoing coil embolization for UIAs, if the thromboembolic risk to the parent artery is not very high, it would be advisable to combine OACs with clopidogrel alone SAPT. In addition, recent studies have shown that in patients with clopidogrel resistance who are undergoing coil embolization, switching to low-dose potent P2Y12 receptor blockers such as prasugrel or ticagrelor on the basis of the results of platelet function tests can reduce the risk of thromboembolic events without increasing the risk of bleeding [9, 22]. Therefore, although it was not analyzed in this study, even in patients taking OACs, assessing clopidogrel resistance and switching to a low-dose potent P2Y12 receptor blocker according to whether the platelet function test indicates resistance can be more beneficial in reducing both thromboembolic and hemorrhagic risks when employing SAPT.

For patients taking OACs, the type of OAC is an important consideration when predicting the impact of coil embolization, as the type of OAC can affect the occurrence of thromboembolic or bleeding complications. Studies in the cardiac literature have reported that in patients taking warfarin, the risk of thromboembolic complications is similar to that in patients using DOACs, but the risk of hemorrhagic complications is greater [15, 23]. In this study, there was no difference in bleeding risk between warfarin and DOACs, and patients on warfarin tended to have a lower bleeding risk. This is likely due to the small number of patients taking warfarin (22 patients) compared with those taking DOACs (90 patients), as indicated by the wide 95% CI range of the HR. Furthermore, patients on warfarin did not receive additional systematic heparinization during the procedure, whereas for patients on DOACs, systematic heparinization was performed inconsistently among the institutions, potentially affecting the results as well.

This study has several limitations. First, the SAPT group included only 31 patients, fewer than the number of patients in the DAPT group. Additionally, within the SAPT group, when patients were divided into the aspirin and clopidogrel subgroups, only seven patients were included in the aspirin alone subgroup. The limited sample sizes in the SAPT group and the aspirin alone subgroup resulted in a lack of statistical power in the results. Second, only patients who either used or did not use a standard neurostent for the treatment of UIAs were included. Therefore, there is uncertainty in directly applying the findings to cases involving flow diverters or the use of other types of stent therapy for treating intracranial stenosis. Third, resistance to antiplatelet therapy may increase the risk of thromboembolic complications during coil embolization. However, since antiplatelet resistance testing was not performed separately in this study, there may be bias related to antiplatelet resistance. Fourth, only thromboembolic and hemorrhagic complications that occurred for 30 days after the procedure were considered, and no information on the continuation of antiplatelet therapy after stent-assisted coil embolization was retrieved. Therefore, further research is needed to analyze the data of such patients. Fifth, although we conducted a multivariable analysis using variables that could affect the primary and secondary outcomes, a multivariable analysis specifically for thromboembolic events was not feasible because of the low number of events. Consequently, the influence of these factors on the primary outcome may not have been fully captured. Finally, although this is a multicentric study, it is a retrospective, nonrandomized design in which the choice of antiplatelet therapy, OACs or systematic heparinization was determined by the treating physician, and therefore it may introduce heterogeneity or be subject to inherent bias. A prospective, randomized controlled trial directly comparing SAPT and DAPT with larger sample sizes may be necessary in the future to increase the generalizability of the results. A prospective, randomized controlled trial directly comparing SAPT and DAPT with larger sample sizes may be necessary in the future to increase the generalizability of the results.

## Conclusions

With respect to SAPT, DAPT was not associated with a reduction in thromboembolic complications in patients taking OACs who underwent coil embolization, but it was associated with an increase in all bleeding events. Therefore, SAPT may be considered a more practical therapeutic option for patients on OACs undergoing coil embolization for UIAs; however, given the limited sample size of this study, further validation through larger studies is warranted.

## Supplementary Information

Below is the link to the electronic supplementary material.

Supplementary File 1 (DOCX 749 KB)

## Data Availability

The datasets generated during and/or analyzed during the current study are available from the corresponding author on reasonable request.
