# Supplementary material for: Comparison between single and dual antiplatelet therapy in patients on oral anticoagulants undergoing coil embolization for unruptured intracranial aneurysms: a retrospective multicenter cohort study
Source: Neuroradiology. 2025 Nov 18;67(12):3439–48. doi: 10.1007/s00234-025-03844-2 (PMC12847220; doi:10.1007/s00234-025-03844-2)

**Supplementary information**

Comparison between single and dual antiplatelet therapy in patients on oral anticoagulants undergoing coil embolization for unruptured intracranial aneurysms: A retrospective multicenter cohort study

**Supplemental Method 1** Coil embolization procedure

When a patient taking oral anticoagulants (OAC) planned to undergo coil embolization for unruptured intracranial aneurysms (UIAs), patients received daily mono antiplatelet therapy using aspirin or clopidogrel 5 days prior to the procedure, or with dural antiplatelet therapy using both aspirin and clopidogrel. All coil embolization were performed under general anesthesia. Intravenous heparin was either not administered or administered at 1000-3000 IU according to each institution’s protocol after the placement of the femoral arterial sheath. In cases where systematic heparinization was performed, heparin was later infused at an hourly booster dose of 1000 IU with monitoring of the activated clotting time. Initially, coil embolization was attempted without the use of a stent, and for aneurysms with unfavorable configuration, a stent was used. The procedure was conventional in all cases. In this study, only detachable bare platinum coils including the Target (Stryker Neurovascular), MicroPlex (MicroVention), and Axium (Medtronic) coils were used. Although we allowed all available stents, only the Enterprise (Cerenovus), Low-profile Visualized Intraluminal Support [(LVIS), MicroVention)] stent, LVIS Jr. stent (MicroVention), or Neuroform Atlas (Stryker Neurovascular) stents were chosen, per the interventionalist’s preference. All puncture sites were closed using a closure device. After the procedure, patients were observed for 24 hours and discharged the next day if there were no abnormal findings. In cases where a stent was used, antiplatelet therapy was maintained for 3 months after the procedure.

**Supplemental Table 1** Summary of thromboembolic events (primary outcome)

| Variable | Aspirin alone | Clopidogrel Alone | Aspirin with Clopidogrel | | |
| --- | --- | --- | --- | --- | --- |
|  |  |  | 1 | 2 | 3 |
| Aneurysm type | MCA bifurcation, 5.6 mm | MCA bifurcation, 4.9 mm | Vertebral artery, 8.3 mm | Anterior choroidal artery, 3.2 mm | Anterior communicating artery, 6.6 mm |
| Coiling method | Double microcatheter | Stent-assisted | Stent-assisted | Double microcatheter | Stent-assisted |
| OAC type | Ribaroxaban 20mg once a day | Ribaroxaban 15mg once a day | Dabigatran 110 mg twice a day | Edoxaban 30 mg once a day | Apixaban 2.5 mg once a day |
| The underlying conditions that require OAC | Pulmonary embolism | Atrial fibrillation | Atrial fibrillation | Atrial fibrillation | Cerebrovascular disease |
| Thromboembolic event | Intraprocedural thrombosis, MCA occlusion | In-stent thrombosis during coiling | Left facial palsy and motor weakness / 4 h after coiling, embolic event | Right side motor weakness during 5 min / 7 d after coiling, TIA event | Left leg weakness / 13 d after coiling, embolic event |
| Treatment | Intra-arterial tirofiban infusion | Intra-arterial fibrinolytics and tirofiban infusion | Conservative | Conservative | Conservative |
| Infarction area | Right MCA territory infarction | No infarction | Right pontine infarction | No infarction | Right ACA territory infarction |
| Modified Rankin Scale score^*^ | 2 | 0 | 2 | 0 | 3 |
| *ACA*, anterior cerebral artery; *MCA*, middle cerebral artery; *OAC*, oral anticoagulant  ^*^Clinical outcome was evaluated 30-day after coil embolization using the modified Rankin Scale score. | | | | | |

**Supplemental Table 2** Summary of all bleeding events (secondary outcome)^*^

| Case | Aspirin alone, (n = 7) | Clopidogrel alone, (n = 24) | Aspirin with Clopidogrel, (n = 81) |
| --- | --- | --- | --- |
| 1 |  | Intraprocedural rupture (major) | Easy bruising (minimal) |
| 2 |  | Easy bruising (minimal) | Femoral artery pseudoaneurysm (minimal) |
| 3 |  |  | Easy bruising (minimal) |
| 4 |  |  | Femoral puncture site oozing (minimal) |
| 5 |  |  | Gingival hemorrhage (minimal) |
| 6 |  |  | Melena (minimal) |
| 7 |  |  | Vitreous hemorrhage (minimal) |
| 8 |  |  | Intraprocedural rupture (major) |
| 9 |  |  | Easy bruising (minimal) |
| 10 |  |  | Easy bruising (minimal) |
| 11 |  |  | Easy bruising (minimal) |
| 12 |  |  | Easy bruising (minimal) |
| 13 |  |  | Easy bruising (minimal) |
| 14 |  |  | Easy bruising (minimal) |
| 15 |  |  | Easy bruising (minimal) |
| 16 |  |  | Intraprocedural rupture (major) |
| 17 |  |  | Easy bruising (minimal) |
| 18 |  |  | Easy bruising (minimal) |
| 19 |  |  | Easy bruising (minimal) |
| 20 |  |  | Easy bruising (minimal) |
| 21 |  |  | Easy bruising (minimal) |
| 22 |  |  | Easy bruising (minimal) |
| ^*^Parentheses indicate the classification according to the Thrombolysis in Myocardial Infarction bleeding criteria. | | | |

**Supplemental Fig. 1** Kaplan‒Meier curves with confidence bands for the Single and Dual Antiplatelet Therapy Groups.

(A) Thromboembolic events


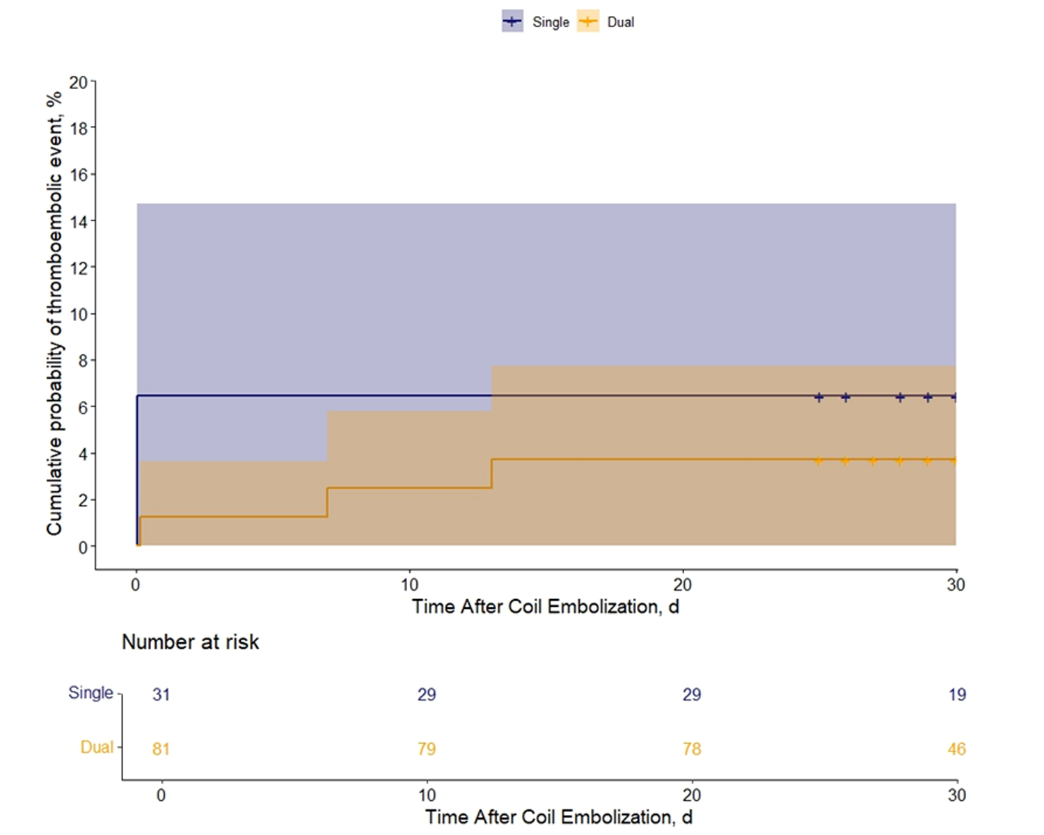


(B) All bleeding events

**
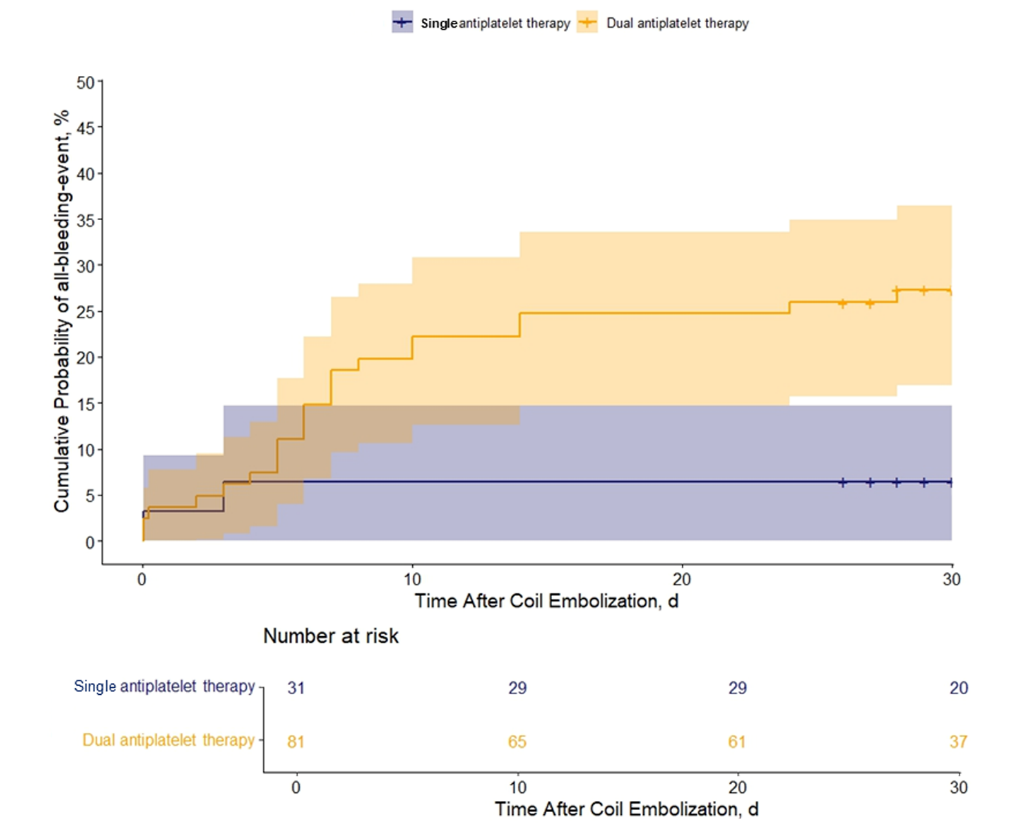
**

**Supplemental Fig. 2** Kaplan‒Meier curves of thromboembolic events in the aspirin monotherapy, clopidogrel monotherapy and dual antiplatelet therapy groups.

(A) Without confidence bands

**
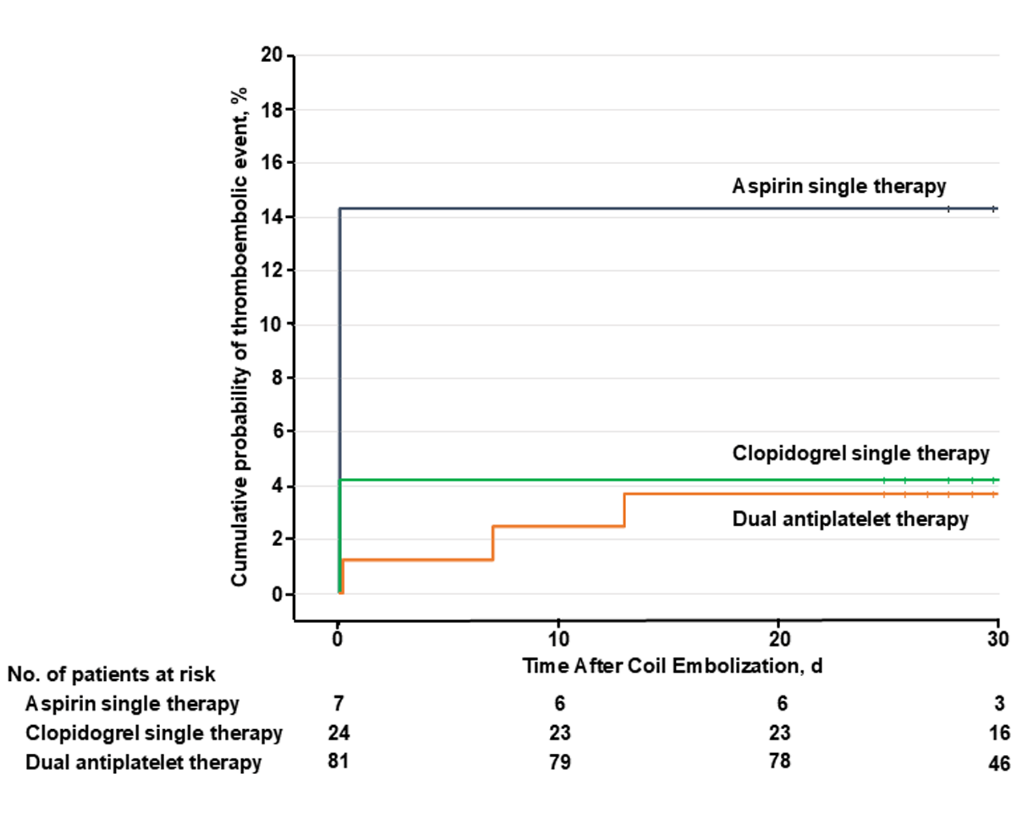
**

(B) With confidence bands


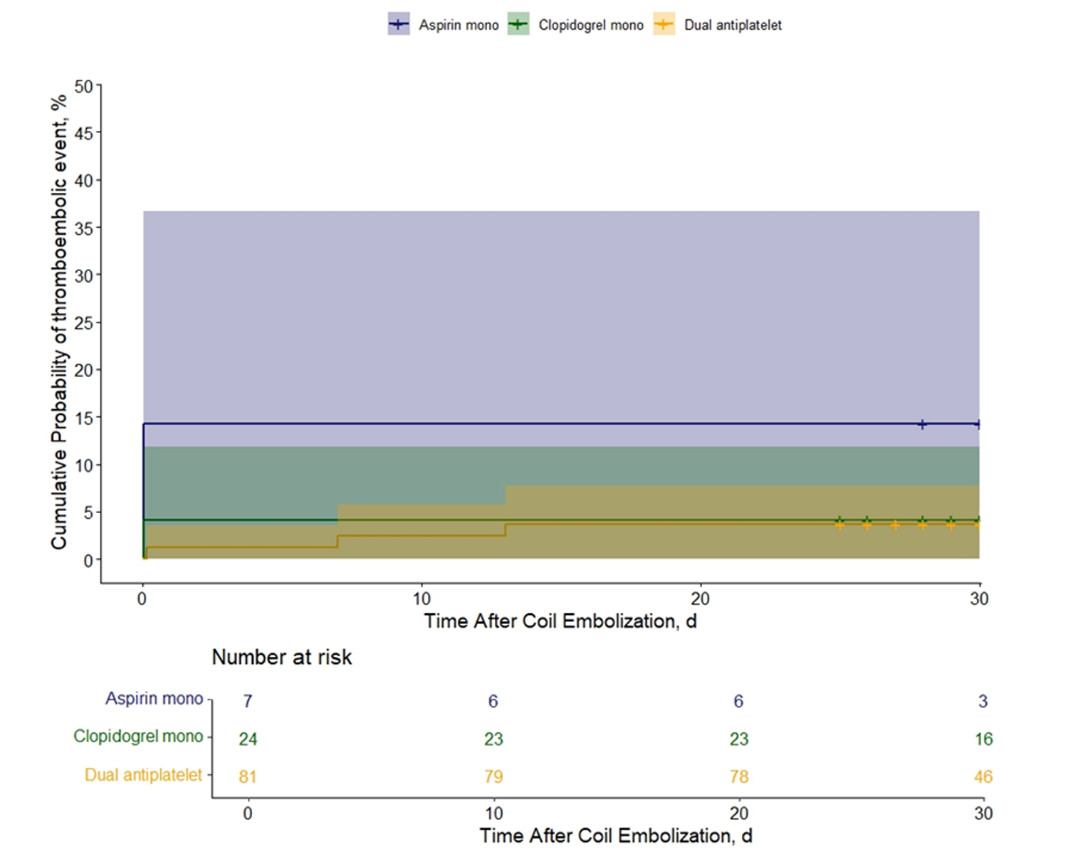

Supplement: Supplementary file 1 — (DOCX 749 KB) [file 234_2025_3844_MOESM1_ESM.docx]
